# Sophorolipid-Based Oligomers as Polyol Components for Polyurethane Systems

**DOI:** 10.3390/polym13122001

**Published:** 2021-06-18

**Authors:** Maresa Sonnabend, Suzanne G. Aubin, Annette M. Schmidt, Marc C. Leimenstoll

**Affiliations:** 1 Macromolecular Chemistry and Polymer Technology, TH Köln, Kaiser-Wilhelm-Allee E39, 51368 Leverkusen, Germany; maresa.sonnabend@gmail.com (M.S.); suzanne_grace.aubin@th-koeln.de (S.G.A.); 2 Department of Physical Chemistry, University of Cologne, Greinstraße 4, 50939 Cologne, Germany; annette.schmidt@uni-koeln.de

**Keywords:** polyurethane, polyol, bio-based, sophorolipid-based polyols, hydroxyl fatty acid based polyols, platform chemicals

## Abstract

Due to reasons of sustainability and conservation of resources, polyurethane (PU)-based systems with preferably neutral carbon footprints are in increased focus of research and development. The proper design and development of bio-based polyols are of particular interest since such polyols may have special property profiles that allow the novel products to enter new applications. Sophorolipids (SL) represent a bio-based toolbox for polyol building blocks to yield diverse chemical products. For a reasonable evaluation of the potential for PU chemistry, however, further investigations in terms of synthesis, derivatization, reproducibility, and reactivity towards isocyanates are required. It was demonstrated that SL can act as crosslinker or as plasticizer in PU systems depending on employed stoichiometry. (*ω*-1)-hydroxyl fatty acids can be derived from SL and converted successively to polyester polyols and PU. Additionally, (*ω*-1)-hydroxyl fatty acid azides can be prepared indirectly from SL and converted to A/B type PU by Curtius rearrangement.

## 1. Introduction

**Figure 1:**
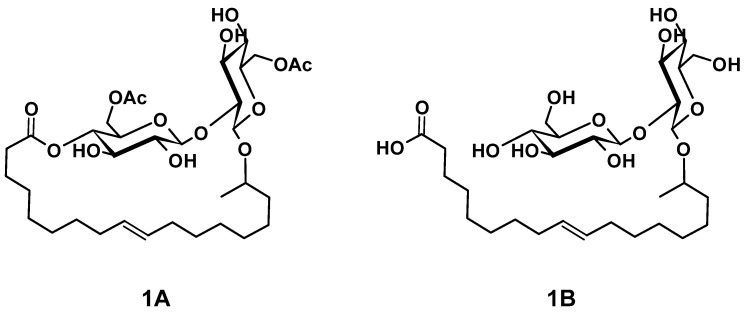
Examples for common types of sophorolipids (SL) comprising unsaturated C18 fatty acid moieties. (**1A**): Acetylated lactonic form (LSL). (**1B**): Deacetylated acidic form (ASL). The number of carbon atoms of the fatty acid chain is usually 16 or 18. The sophorose unit is typically attached to the fatty acid moiety at its second last carbon atom (*ω*-1) or at its terminal carbon atom (*ω*, structure not shown).

The main components of well-known and very versatile polyurethanes (PU) [1,2] are polyisocyanates and polyols [3,4]. The morphology and thus the properties of PU are mainly determined by the intrinsic structure of the polyol component(s) since their content exceeds typically 60 wt.% of the PU [5,6,7]. The polyols most commonly utilized are polyether, polyester, and polycarbonate polyols [5,8]. All types of polyols are usually produced by means of petrochemical resources. Considering the need for a sustainable economic activity [9], the development of polyols based on renewable resources and the research on their applicability for PU systems are of increased significance [10,11,12,13,14,15,16,17,18]. Among bio-based polyol platforms, sophorolipids (SL) are well documented and show high potential to provide a versatile toolbox for polyol building blocks [19,20,21,22,23,24,25,26]. Specific advantages of SL are their non-pathogenic production organisms, possessing high productivity and an efficient rate of substrate conversion [21]. SL consist of sophorose, a hydrophilic di-glucose, coupled to a hydrophobic hydroxyl fatty acid (HFA) by a glycosidic bond (Figure 1(**1B**)). The resulting SL is amphiphilic and thus readily utilized as bio-based surfactant in, e.g., surface technology [24,27,28]. The most effective organism to produce SL is *bombicola*, which is capable of precipitating up to 400 g L^−1^ SL into the fermentation medium [20]. Moreover, structural variation is feasible by feeding different lipid derivatives with C16 and C18 fatty acids, which incorporate in particularly good yields [29,30]. The most common SL derivatives are lactonic SL (LSL) and acidic SL (ASL), as depicted in Figure 1 [24,31].

Dihydroxyl alkyl glycosides are structurally similar to SL and were already converted successfully to PU by reaction with diisocyanates [14,32]. In these procedures, the double bond of the fatty acid moiety is transformed into a vicinal diol structure, which is subsequently converted to PU by reaction with the isocyanate (NCO) groups of isophorone diisocyanate (IPDI). Consequently, SL should perform similarly to give PU. Moreover, LSL has six hydroxyl (OH) groups (two acetylated primary OH groups and four secondary OH groups) and ASL seven OH groups (two acetylated primary OH groups and five secondary OH groups) per molecule. This means that a successive conversion with (di)isocyanates is generally possible because the secondary OH groups are readily available for the reaction with NCO groups, whereas the primary OH groups become available after, e.g., saponification [33]. The complete deacetylated ASL should also allow a certain selectivity since primary OH groups react with NCO groups about three times faster than secondary OH groups [34,35,36,37,38]. Utilizing appropriate reaction conditions, primary OH groups can purposely be consumed first in order to produce linear and thus thermoplastic PU [38,39,40,41,42,43]. In contrast, complete conversion of all OH groups leads to significant crosslinking and therefore to a thermosetting material. Considering the Flory–Stockmayer relation [44], the degree of crosslinking and thus the nature of the thermosetting material can be tailored to a certain extent, e.g., by appropriate choice of the NCO/OH ratio (index) [44,45,46]. In summary, SL provide a high number of potential reaction sites with different reactivity toward NCO groups and should therefore be suited as crosslinking agent and potentially as a linear building block [32]. Additionally, SL are suited as internal emulsifier in PU dispersion technology because of their amphiphilic nature [47,48,49].

HFA form renewable resources like castor oil, which can be converted to polyester polyols or directly to PU [17,50,51,52]. Typically for such HFA is that the OH group is localized in the center of the molecule. The resultant polyester or PU systems consequently contain dangling chains that reduce crystallinity and glass transition temperature [52,53]. In contrast to that, *ω*- or (*ω*-1)-HFA yield systems without side chains and with lower ester- or urethane group concentrations. Therefore, dangling chains are not expected and lipophilicity is increased suggesting enhanced hydrolysis resistance and improved interactions to hydrophobic surfaces [54]. *ω*- or (*ω*-1)-HFA with varying chain lengths can be obtained by fragmentation of SL [26] and successively converted chemically [55] or enzymatically [56,57,58,59,60] to yield polyesters via A/B self-condensation. Note that successive conversion with diisocyanates to high molecular weight PU requires two-fold OH termination, which can be accomplished, e.g., by initiation with or addition of suitable diols [55,61]. Additionally, the acid moiety of HFA can be converted to azide in order to accomplish Curtius rearrangement [62] yielding A/B type polyurethanes [51,63,64,65,66,67,68].

The present work shows that LSL can act as crosslinker or as plasticizer in PU systems depending on employed stoichiometry. (*ω*-1) HFA-based polyester polyols and (*ω*-1) HFA-based PU systems are feasible; however, low amounts of glucose impurities seem to limit the versatility of the reaction since there is a substantial extent of crosslinking and branching. Additionally, we demonstrate the fundamental access to A/B type PU systems by applying Curtius rearrangement on (*ω*-1) HFA azides obtained from LSL, confirming the Curtius approach to A/B type PU systems of other groups [51,64,65,66,67,68,69].

## 2. Materials and Methods

### 2.1. Materials

1,4-Dioxane (pure), dimethyl sulfoxide (DMSO, ≥99%), oxalyl chloride (for synthesis), and tetrabutylammonium hydroxide (in 2-propanol/methanol, TitriPUR) were from Merck Millipore. 4-*tert*-Butylcatechol (99%), *N*-methylpyrrolidin-2-one (NMP, for synthesis), and sodium azide (NaN_3_, >99%) were from Acros Organics. 12-Hydroxystearic acid (95%), sodium sulfate (Na_2_SO_4_, anhydr., 99%), and *tert*-butyl methyl ether (MTBE, ≥99.9%) were from Alfa Aesar. 1-Hexylisocyanate (HIC, 97%), dibutylamine (99.5%), phenylisocyanate (PIC, for synthesis), and tin(II) chloride (SnCl_2_, 98%) were from Sigma Aldrich. 1,6-Hexanediol (for synthesis), 1,6-hexamethylenediisocyanate (HDI, >98%), 2-butanone (MEK, >99%), 2-propanol (≥99%), acetonitrile (≥99.95%), *N,N*-dimethylformamide (DMF, ≥99.8%), hydrochloric acid (HCl, 1 M, TitriPUR), potassium hydroxide (KOH, 0.1 M in methanol, TitriPUR), and sodium chloride (NaCl, ≥99%) were from VWR Chemicals. Deuterated chloroform (CDCl_3_ with 0.03% TMS, 99.8 atom%D) and deuterated THF (THF-d_8_ with 0.03% TMS, 99.8 atom%D) were from Deutero. Acetone (≥99%), cyclohexane (≥99.5%), chloroform (CHCl_3_, >99%), dichloromethane (CH_2_Cl_2_, for analysis), diethyl ether (≥99.5%), ethanol (abs.), ethyl acetate (for analysis), methanol (HPLC grade), petroleum ether (for analysis), tetrahydrofuran (THF, HPLC grade), and toluene (≥99.9%) were from Fisher Chemical. Sodium hydroxide (NaOH, pure) was from Bernd Kraft. *n*-Hexane (≥95%) was from Carl Roth. Dibutyltindilaurate (DBTDL, technical grade) and poly(1,4-butylene)adipate (PBA, technical grade, M_n_ = 2250 g mol^−1^) were kindly provided by Covestro Deutschland AG. SL (**1A**) (LSL) (Figure 1) was synthesized and purified according to the methods published elsewhere and provided by Zerhusen and Schörken [19,25,27]. 1,6-Hexanediol and PBA were dried for at least 2 h under reduced pressure, and kept at elevated temperature (50 mbar, 80 °C) before use. All other chemicals were used as received or purified applying established and well-known procedures [70].

### 2.2. Measurements and Equipment

Size exclusion chromatography (SEC) was carried out on a PSS Polymer SECcurity system based on Agilent 1260 hardware modules equipped with SECcurity isocratic pump, vacuum degasser, refractive index and UV-Vis detector (254 nm), column oven, and a standard auto sampler. A styrene-divinylbenzene copolymer column (SDV linear XL (100–3,000,000 Da)) with 5 µm particle size and 1000 Å porosity was calibrated with polystyrene ReadyCal Kit (PSS Polymer) standards. Measurements were carried out in THF at 30 °C with a flow rate of 1.0 mL min^−1^. Integration of the signals was performed via software package “WinGPC Unity” from PSS Polymer.

^1^H- and ^13^C-NMR spectra were recorded using a Bruker Ascend 400 spectrometer (400 MHz) at room temperature using CDCl_3_ or THF-d_8_ as solvent (sample conc. = 0.05 mg µL^−1^). Tetramethylsilane (TMS) was used in all experiments as internal standard.

ATR-IR spectra were recorded using a Bruker Platinum-ATR equipped with a MIR-RT-DLaTGS detector and a KBr radiation plate. OPUS software of Bruker Optic GmbH was used for data handling. Spectra were recorded at room temperature, applying 24 scans, and a resolution of 4 cm^−1^. Background spectra were recorded in ambient atmosphere prior to each measurement. For reaction monitoring, inline-IR-spectroscopy was used applying Thermo Fisher Nicolet iS 50-spectrometer equipped with a probe coupler and the ZnSe-ATR-tube FlexiSpec^®^ from art photonics GmbH, Berlin. HgCdTe detector was cooled with liquid N_2_. The software “Macros Basic” enabled 20 automated scans per minute in a resolution of 2 cm^−1^. Background spectra were recorded in dry N_2_ prior each measurement. “OMNIC” software was used to calculate peak areas.

Differential scanning calorimetry (DSC) was conducted applying Q2000 DSC from TA instruments consisting of an auto sampler and a RCS90 cooling unit calibrated by an indium standard. Samples were placed in Tzero aluminum crucibles. SL containing samples were heated to 150 °C and cooled to −60 °C twice using a rate of 10 K min^−1^. The results of the second cycle were used for evaluation.

X-ray diffraction (XRD) was conducted with a Bruker D2 Phaser 2nd Gen equipped with a Cu tube (*λ* = 1.54184 Å), fixed slit 0.4 mm, 4° grid, and a Lynx 1D Modus detector as single measurements in order to obtain data for crystallinity (angle of incidence: 5–80°; rotation: 0–15 rpm; increment: 0.03°; time per step: 0.02 s; scan type: Coupled Two Theta Theta; scan modus: Continuous PSD fast). Samples were applied as films or grinded powder. For evaluation, Bruker’s Diffrac EVA software was used.

High-performance liquid chromatography (HPLC) was conducted using Shimadzu Nexera XR equipped with a BM-20 A communications bus module, two LC-20 AD XR, a SIL-D0ACXR auto sampler, a column oven, and a VWR-ELSD 80 detector. Conditions: N_2_; 3.5 bar; 40 °C; column: 4.6 mm × 250 mm, 5 µM C18 RP La Chrom II (Hitachi, Tokio, Japan); flow rate: 1.0 m min^−1^; 30 °C; solvent: acetonitrile/water (50 vol.%:50 vol.% for 25 min. to 99 vol.% acetonitrile linearly within 60 min.); samples: 2.5 mg mL^−1^ in THF.

Acid number, hydroxyl number and the content of NCO groups were determined according to DIN EN ISO 660 2009, DIN 53240-2 (ASTM E1899-08), and DIN EN ISO 11909 2007, respectively. Automated titration was conducted with a TitroLine 7000 titration unit from SI Analytics.

Shore A hardness was measured five times for each sample according to DIN EN ISO 868 applying SAUTER HBA 100-0 and SAUTER TI-A0 with a contact pressure of 5 kg and calibrated with Durometer Test Block Kit AHBA-01 (SAUTER). Tensile tests were conducted according to DIN EN ISO 527-1/-2 (ASTM D 638) on a Shimadzu Autograph AG-X plus tensile testing machine (50 mm min^−1^). TrapeziumX (Shimadzu, Kyoto, Japan) software was used for evaluation. The samples were polymer films produced by casting the liquid polymer (2000 µM) on a Teflon plate with a squeegee.

The preparation of SL solutions was as follows: 100 mg (0.145 mmol) of (**1A**) (LSL) or 90.5 mg (0.145 mmol) (**1B**) (ASL) was transferred at room temperature to 2 mL of solvent and the solubility evaluated optically.

### 2.3. Synthesis of 17-Hydroxyoctadec-9-Ene Acid ((ω-1) HFA)

**Figure 2:**
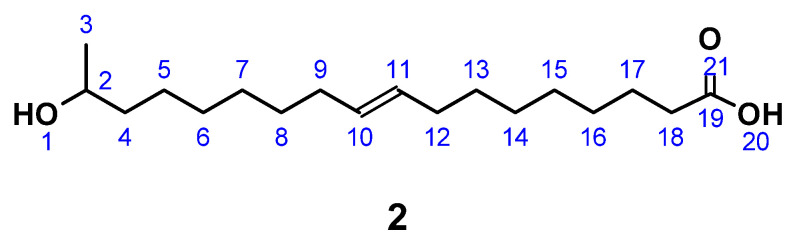
17-Hydroxyoctadec-9-ene acid ((*ω*-1) HFA) (**2**).

(*ω*-1) HFA (**2**) (Figure 2) was produced by a modification of published procedures in absence of carcinogenic dioxane, but at the expense of reaction time [26,71]. A typical protocol was as follows. LSL (**1A**), derived from *starmerella bombicola* (97% purity) [19], was dissolved in 5 M NaOH solution. To this solution, a further 5 M NaOH was added dropwise until a constant pH was reached. After pH adjustment to 3.5 with diluted HCl, the intermediate (**1B**) was crystallized at 7 °C and purified by lyophilization to give a white powder. C_30_H_54_O_13_; M = 622.75 g mol^−1^; SEC: M_n_ = 530 g mol^−1^; M_w_ = 660 g mol^−1^; PDI = 1.3; yield: 89.2%; FT-IR (ATR) *ν*/cm^−1^: 3324 (m), 2921 (m); 2851 (m); 1726 (m); 1559 (w); 1155 (m); 1070 (s); 1019 (s); 894 (m); 643 (m); 511 (m); HPLC: 10 min.

A total of 5.0 g (0.008 mol) (**1B**) was placed in 100 mL round-necked flask and dissolved under stirring in 50 mL 1 M HCl. The reaction proceeds at 80 °C, turning the clear liquid to a turbid solution comprising particles and further to a biphasic system with yellowish, oily droplets. The reaction progress was monitored by HPLC. After completion of hydrolysis, the pH was adjusted to 3.5 by addition of diluted NaOH. The product was purified by solvent extraction (distilled H_2_O/CHCl_3_) and removal of excessive solvent in vacuo. The product (**2**) accumulated as yellowish oil in 83.5% yield. Since dimers and oligomers were identified, (**2**) was stirred in 5 M NaOH at 80 °C for a further 16 h. Repeated solvent extraction and removal applying the same conditions yielded a brownish oil.

C_18_H_34_O_3_; M = 298.47 g mol^−1^; OH number = 240; acid number = 86.1; yield: 54.3%; FT-IR (ATR) *ν*/cm^−1^: 3393 (w), 3004 (w), 2924 (s), 2853 (s), 1709 (s), 1460 (m), 1375 (m), 1243 (m), 1191 (m), 1081 (m), 754 (s); HPLC: 40 min. (monomer), 89 min. (dimer); ^1^H NMR (400 MHz, CDCl_3_, δ): 5.40–5.28 (m, 2H; H10, H11), 3.81 (m, 1H; H2), 2.34 (t, *J* = 7.5 Hz, 2H; H18), 2.07–1.96 (m, 4H; H9, H12), 1.69–1.52 (m, 4H; H4, H17), 1.43 (d, *J* = 3.7 Hz, 4H; H8, H13), 1.37–1.22 (m, 12H; H5 – 7, H14–16), 1.19 (dd, 3H; H3); ^13^C NMR (101 MHz, CDCl_3_, δ): 178.66 (C19), 129.85 (C10, C11), 68.33 (C2), 39.25 (C4), 34.78 (C18), 29.69–28.99 (C6–C8, C13–C16), 27.19 (C9, C12), 25.75 (C5), 24.73 (C17), 23.43 (C3).

### 2.4. Synthesis of (ω-1) HFA Based Polyester Diol

**Figure 3:**
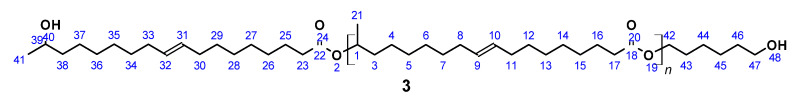
(*ω*-1) HFA based polyester diol (**3**).

OH-terminated (*ω*-1) HFA-based polyester diol (**3**) (Figure 3) was prepared according to a published procedure [61]. A total of 9.0 g (0.03 mol) (*ω*-1) HFA (**2**) and 0.7 g (0.006 mol) 1,6-hexanediol was introduced in a 100 mL flask equipped with a Vigreux column, thermometer, and distilling link. The reaction mixture was heated to 200 °C within 1 h. Reaction water was removed by distillation. In contrast to the published procedure, no catalyst (SnCl_2_) was used. Instead, 7.0 mg (0.042 mmol) 4-*tert*-butylcatechol was added in order to prevent undesired reactions at the double bond. After 24 h, the reaction was stopped, leaving a highly viscous and sticky product, which was partially soluble in THF.

SEC (soluble fraction): M_n_ = 2370 g mol^−1^ (M_n,theo_ = 2250 g mol^−1^), M_w_ = 99,700 g mol^−1^, PDI = 42.1; acid number = 2.8; yield: not determined; FT-IR (ATR) *ν*/cm^−1^: 3350 (m), 2930 (s), 2858 (s), 1726 (s), 1460 (m), 1374 (m), 1174 (m), 1055 (s), 727 (w); ^1^H NMR (400 MHz, THF-d_8_, δ): 5.33 (t, *J* = 4.8 Hz, 4H; H9, H10, H31, H32), 4.89–4.78 (m, 1H; H1), 4.15–4.05 (m, 2H; H42), 4.01 (t, *J* = 6.6 Hz, 3H; H39, H47), 2.31–2.16 (m, 4H; H17, H23), 2.03 (m, 8H; H8, H11, H30, H33), 1.72–1.45 (m, 12H; H3, H16, H25, H38, H43, H46), 1.45–1.20 (m, 49H; H4–H7, H12–15, H21, H26–29, H34–H37; H44, H45), 1.16 (d, *J* = 6.2 Hz, 1H; H41); ^13^C NMR (101 MHz, THF-d_8_, δ): 172.30 (C18, C22), 129.54 (C9, C10, C31, C32), 69.85 (C1), 66.87 (C39), 63.47 (C47), 35.92 (C38), 34.04 (C3), 33.66 (C17, C23), 29.69 (C36), 29.22–28.62 (C5–C8, C12–C14, C27–C29, C34–C36), 27.03 (C8, C11, C30, C33), 25.61–24.74 (C16, C25, C37, C45), 23.95 (C41), 19.35 (C21).

### 2.5. 12-Hydroxystearic Acid Based Polyesterdiol 

**Figure 4:**
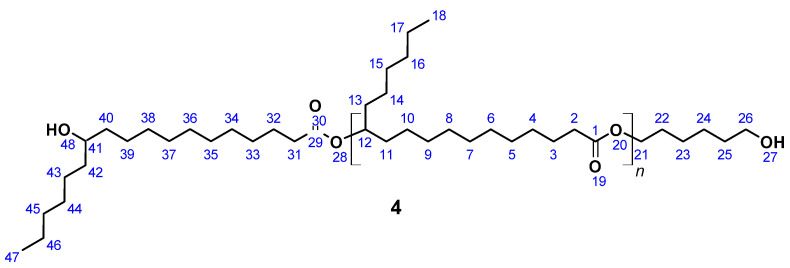
12-Hydroxystearic acid based polyesterdiol (**4**).

OH-terminated 12-hydroxystearic acid based polyesterdiol (**4**) (Figure 4) was prepared according to a published procedure [61]. A total of 5.0 g (16.7 mmol) 12-Hydroxystearic acid and 0.41 g (3.5 mmol) 1,6-hexanediol was introduced in a 20 mL flask equipped with a Vigreux column, thermometer, and distilling link. The reaction mixture was heated to 200 °C within 1 h. Reaction water was removed by distillation. After 24 h, SnCl_2_ was added in order to complete the conversion. After another 24 h, vacuum was applied to remove water residues leaving a highly viscous liquid as the product.

SEC: M_n_ = 2210 g mol^−1^ (M_n,theo_ = 2250 g mol^−1^), M_w_ = 5800 g mol^−1^, PDI = 2.6; acid number = 2.1; T_g_ = −20 °C; yield: quantitative; FT-IR (ATR) *ν*/cm^−1^: 3350 (w), 2922 (s), 2852 (s), 1731 (s), 1464 (m), 1376 (w), 1174 (m), 1100 (w), 723 (w); ^1^H NMR (400 MHz, THF-d_8_, δ): 4.85 (m, *J* = 6.2 Hz, 1H; H12), 4.01 (t, *J* = 6.6 Hz, 2H; H21), 3.41 (s, 1H; H41), 3.25 (s, 2H; H26), 2.24 (m, *J* = 7.4, 3.1 Hz, 4H; H2, H31), 1.59 (m, *J* = 8.3, 5.2, 4.4 Hz, 8H; H3, H22, H25, H32), 1.56–1.47 (m, 16H; H4, H11, H13, H23, H24, H33, H40, H42), 1.40–1.19 (m, 44H; H4–H10, H14–H17, H33–H39, H43–H46), 0.89 (t, *J* = 6.5 Hz, 6H; H18, H47); ^13^C NMR (101 MHz, THF-d_8_, δ): 172.05 (C1, C29), 73.03 (C12), 70.34 (C41), 65.98 (C21), 63.45 (C26), 37.91 (C40, C42), 34.21 (C31), 34.03 (C11, C13), 31.79 (C23, C45), 29.90–28.63 (C4–C9, C15, C33–C37, C44), 25.80 (C3, C24, C32), 24.90 (C17, C46), 13.47 (C18, C47).

### 2.6. Synthesis of 17-Hydroxyoctadec-9-Enoyl Azide ((ω-1) HFA Azide)

**Figure 5:**
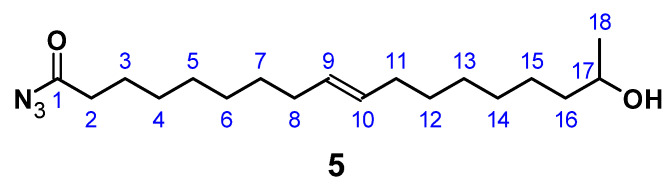
17-Hydroxyoctadec-9-enoyl azide ((*ω*-1) HFA azide) (**5**).

The synthesis of (**5**) (Figure 5) follows a modification and combination of several published procedures in order to improve reaction time and yield [72,73]. In a 100 mL flask, 0.75 g (2.50 mmol) (*ω*-1) HFA (**2**) was dissolved in 15 mL dry CH_2_Cl_2_. To this solution, 0.25 mL (3.0 mmol) oxalyl chloride and 2.30 mL (0.03 mmol) DMF was added, and the reaction mixture was stirred at room temperature for 1 h. Then the solution was cooled to 0 °C and 0.65 mg (10 mmol) of an aqueous NaN_3_ solution was added dropwise. The resulting mixture was stirred at 0 °C for 3 h. The organic phase was extracted by 3 × 30 mL CHCl_3_. The combined organic layers were washed with 40 mL brine, dried over anhydrous Na_2_SO_4_, and concentrated under reduced pressure. The product accumulated as a yellow oil.

C_18_H_33_N_3_O_2_; M = 323.47 g mol^−1^; yield: 49%; FT-IR (ATR) *ν*/cm^−1^: 3335 (w), 2926 (s), 2854 (m), 2158 (w), 1704 (s), 1658 (s), 1368 (m), 1288 (w), 1168 (m), 1064 (m), 976 (w), 832 (w), 721 (w); ^1^H NMR (400 MHz, CDCl_3_, δ): 5.34 (t, *J* = 11.71, 7.3 Hz, 2H; H9, H10), 4.83–4.71 (m, 1H; H-OH), 3.72 (m, 1H; H17), 2.31 (t, *J* = 7.53 Hz, 2H; H2), 2.01 (m, 4H; H8, H11), 1.68–1.57 (m, 4H; H3, H16), 1.49–1.37 (m, 6H; H7, H15, H17), 1.18 (m, 10H; H4–H6, H13, H14), 1.18 (d, 3H; H18); ^13^C NMR (75 MHz, CDCl_3_, δ): 178.4 (C1), 130.0 (C9, C10), 68.2 (C17), 39.3 (C16), 29.8 (C2), 29.2 (C4), 29.1 (C7, C12), 29.0 (C5, C6, C13), 28.9 (C5, C11), 27.2 (C14), 25.6 (C15), 25.2 (C3), 23.7 (C18).

### 2.7. Preparation of Urethanes

#### 2.7.1. LSL and PBA-Based Urethanes

**Figure 6:**
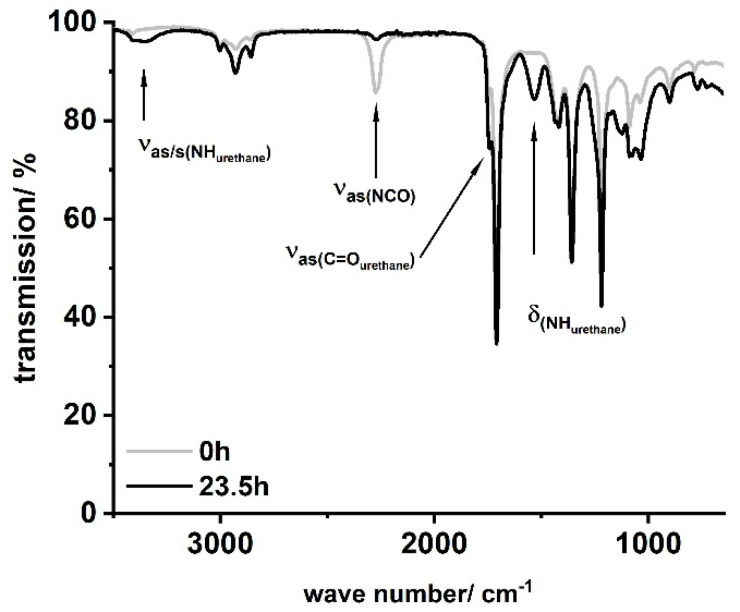
Typical FT-IR spectra for products obtained by the reaction of LSL or PBA with mono- or diisocyanates according to Table 1. Sample shown: LSL-HDI_1.1_.

In a typical procedure, LSL ((**1A**), M = 694.44 g mol^−1^, *f*_OH_ = 4) or PBA (*f*_OH_ = 2) was placed in a three-necked flask, dissolved in 50–80 mL acetone, and heated to the desired temperature shown in Table 1. The corresponding amounts of HIC, PIC, or HDI and DBTDL were added to the solution and the reaction was monitored by inline IR and NCO titration. Residual amounts of diisocyanate were quenched with excessive MeOH prior to further investigations. Figure 6 shows characteristic IR spectra obtained applying this procedure exemplarily for the sample LSL-HDI_1.1_.

#### 2.7.2. Synthesis of PU Based on (*ω*-1) HFA-Based Polyesterdiol and 12-Hydroxystearic Acid Based Polyesterdiol 

(*ω*-1) HFA-based polyesterdiol (**3**) was dissolved in THF for 7 d. After this period, solubility was complete and M_n_ was measured to be 1561 g mol^−1^. A total of 25 mL THF solution containing 3.5 g (2.3 mmol) (**3**) was heated to 60 °C and 500 ppm DBTDL and 0.58 g (3.5 mmol) HDI was added. According to NCO titration, the reaction was completed after 16 h, cooled to room temperature and the solvent removed under reduced pressure. Residual HDI was quenched by 0.1 M dibutylamine solution in acetone leaving a dark brown rubberlike product.

SEC: M_n_ = 3530 g mol^−1^ (M_n,theo_ = 3406 g mol^−1^), M_w_ = 772,330 g mol^−1^, PDI = 218.7; T_g_ = −43 °C; FT-IR (ATR) *ν*/cm^−1^: 3333 (w), 3002 (w), 2925 (s), 2854 (s), 1732 (s), 1621 (m), 1533 (s), 1459 (m), 1373 (m), 1234 (s), 1172 (s), 726 (m), 590 (w).

12-Hydroxystearic acid based polyesterdiol (**4**) was dissolved in THF for 7 d. After this period, M_n_ was measured to be 1862 g mol^−1^. A total of 25 mL THF solution containing 3.5 g (2.3 mmol) (**4**) was heated to 60 °C and 500 ppm DBTDL and 0.47 g (2.8 mmol) HDI was added. According to NCO titration, the reaction was completed after 16 h, cooled to room temperature, and the solvent removed under reduced pressure. Residual HDI was quenched by 0.1 M dibutylamine solution in acetone, leaving a dark brownish oil as product.

SEC: M_n_ = 4170 g mol^−1^ (M_n,theo_ = 5209 g mol^−1^), M_w_ = 11,530 g mol^−1^, PDI = 2.8; T_g_ = −43 °C; FT-IR (ATR) *ν*/cm^−1^: 3333 (w), 3002 (w), 2925 (s), 2854 (s), 1732 (s), 1621 (m), 1533 (s), 1459 (m), 1373 (m), 1234 (s), 1172 (s), 726 (m), 590 (w).

#### 2.7.3. Preparation of A/B Type PU 

**Figure 7:**
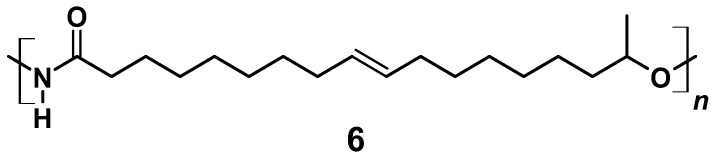
A/B Type PU (**6**) derived by Curtius rearrangement of azide (**5**).

In a 100 mL round-necked flask, 0.80 g (2.5 mmol) 17-Hydroxyoctadec-9-enoyl azide (**5**) was dissolved in 1 mL THF under N_2_ atmosphere. Curtius rearrangement and successive polymerization was started applying different temperatures and allowed to proceed for different periods of time (for details see Section 3). The resulting A/B type polymer (**6**) is depicted in Figure 7.

#### 2.7.4. Ternary Urethane Systems

For kinetic investigations, 16.42 g (7.0 mmol) PBA and 5 g (7.0 mmol) LSL were dissolved in 80 mL acetone. At 50 °C, 6.1 g (0.048 mol) HIC or 5.7 g (0.048 mol) PIC was added. The reaction was immediately started by addition of 500 ppm DBTDL and monitored by NCO titration.

For variation of the polyol composition, corresponding amounts of LSL and PBA (Table 2) were placed in an appropriate flask, dissolved in acetone, and heated to 50 °C. After addition of HDI and 500 ppm DBTDL, the reaction was started and monitored by NCO titration. After completion of the reaction, the product was cast on a Teflon plate applying a squeegee (2000 µM) and dried at room temperature.

## 3. Results and Discussion

### 3.1. Sophorolipids as Polyol Component for PU Systems

#### 3.1.1. Solubility Studies

Lactonic SL (LSL, Figure 1(**1A**)), and acidic SL (ASL, Figure 1(**1B**)) can be produced in good yields and with a purity of at least 97% [19,25,27]. The melting points of LSL and ASL ranges between 55 °C and 65 °C. Above this temperature range, the SL remain highly viscous. The reaction with isocyanates in bulk is thus quite challenging, which is why the utilization of an appropriate solvent becomes highly favorable. The solubility of LSL and ASL in selected solvents is listed in Table 3.

LSL is soluble in several solvents, particularly in acetone, MEK, and THF, all being solvents commonly used in PU synthesis. In contrast to that, ASL shows poor solubility in these solvents. In fact, solubility is sufficient in distilled water and methanol, both being solvents with relatively high polarity. This is assumed to be the result of the higher intrinsic polarity of ASL due to the additional OH- and COOH- functionalities. Note however that acceptable solubility is also measured using less polar DMF and DMSO, suggesting a more complex solubility behavior that is determined not alone by polarity.

Water and methanol are well-known to be inappropriate solvents to study the conversion of polyols with isocyanates since they react themselves with isocyanates forming urea and urethane groups [1,3]. DMF and DMSO are improper solvents as well because DMF reacts with isocyanates forming amidines and biurets [74,75] and DMSO yields sulfide esters by reaction with the carboxyl group of ASL [76]. Hence, the reaction of SL with isocyanates in solution is studied applying LSL.

#### 3.1.2. Reaction of LSL with Monoisocyanates

**Figure 8:**
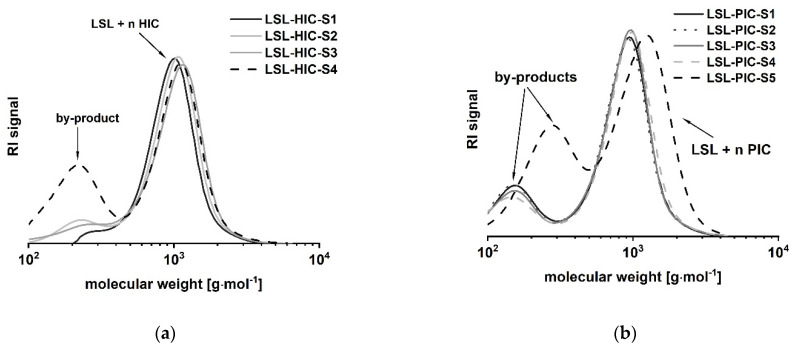
Size exclusion chromatograms of products from the reaction of LSL with (**a**) 1-isocyanatohexane (HIC); (**b**) phenylisocyanate (PIC) applying different reaction conditions (Table 1).

LSL comprises four secondary OH groups (Figure 1(**1A**)). The extent of the conversion of the OH groups can be investigated by reaction with monofunctional isocyanates HIC and PIC. Assuming complete conversion, the reactions of LSL with HIC or PIC should yield products with M_n,theo,LSL-HIC_ = 1197 g mol^−1^ and M_n,theo,LSL-PIC_ = 1165 g mol^−1^, respectively. As shown in Figure 8, the experimental molecular weights are determined to be in the range of about 1000 g mol^−1^ for all systems applying slight excess of isocyanate. This means that on average, 2.5 OH groups of the LSL reacted with the isocyanate. In contrast to this, a five-fold excess of PIC must yield a product with M_n,theo,LSL-PIC_ ≈ 1155 g mol^−1^, thus indicating complete conversion. In addition to the urethane products, by-product formation is observed (Figure 8). Residual isocyanates could not be detected in a significant amount. For the PIC systems with low isocyanate excess, the molecular weight of the by-product is about 140 g mol^−1^ and can be assigned to phenyl urea (M_n_ = 136.15 g mol^−1^) being an impurity of PIC. It is well-known that monoisocyanates dimerize to form uretdiones [79,80]. According to ^13^C-NMR analysis and conversion-time-investigations (results not shown) [81], the signals at ca. 250 g mol^−1^ correspond to the uretdione dimers of HIC (M_n,theo,HIC-uretdion_ = 254.37 g mol^−1^) and PIC (M_n,theo,PIC-uretdion_ = 238.24 g mol^−1^), respectively. The dimerization explains thus excellently the incomplete conversion of OH groups of LSL applying minor excess of isocyanate. With large excess, however, uretdione formation becomes prominent, but remains insufficient to prevent completion of the reaction.

#### 3.1.3. Reaction of LSL with Diisocyanates

##### Stoichiometric Influence

**Figure 9:**
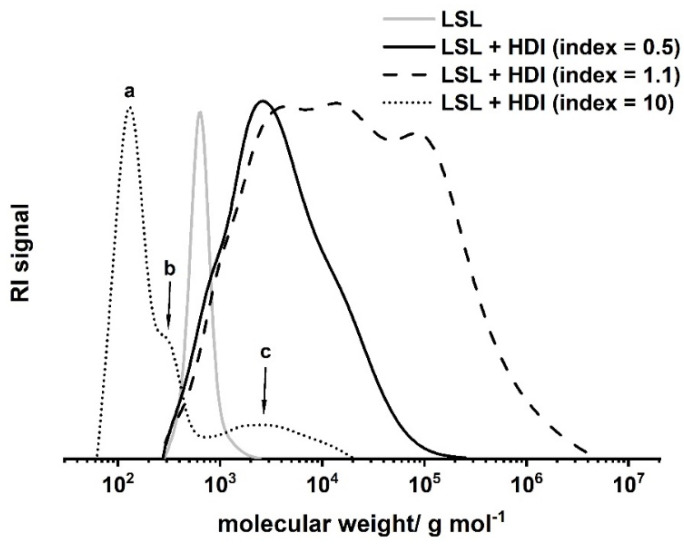
Size exclusion chromatograms of products from the reaction of LSL with HDI applying different NCO/OH ratios (index). Reaction conditions: solvent = acetone; T = 50 °C; c_DBTDL_ = 500 ppm. Peak a corresponds to residual HDI, b to mono- and diurethanes from quenching residual HDI with excessive MeOH, and c to oligomers/polymers from the reaction of LSL with 10-fold excess of NCO groups.

To produce high molecular weight polyurethanes, LSL has to be converted with isocyanates with a functionality *f* of at least two. In contrast to the reaction with monofunctional isocyanates, the reaction with HDI does not lead to measurable uretdione formation. However, because *f*_LSL_ is larger than two, crosslinking is substantial and thus the point of gelation must be considered when different stoichiometric NCO/OH ratios (indices) are applied. The point of gelation can be calculated applying the Flory–Stockmayer relation (1) [44,45,46].

(1) $${\left(p_{OH}\times p_{NCO}\right)}_{gel}={\left(f_{OH}-1\right)}^{-1}{\left(f_{NCO}-1\right)}^{-1}$$

with *p*_OH_ and *p*_NCO_ = conversion of OH and NCO groups, respectively, and *f*_OH_ and *f*_NCO_ = functionality of the OH and the NCO component. For the LSL/HDI system, the point of gelation is calculated to be 1/3. Thus, gelation is to be expected within an index range of 0.33 to 3.03. A series of experiments applying different indices allows detailed inspection of the reaction of LSL with HDI (Figure 9). The reaction of LSL with 10-fold excess of HDI leads to a product mixture that is still soluble in THF. SEC shows the nature of the components of this mixture (Figure 9, dotted line). Peak c (M_n_ = 2190 g mol^−1^) relates to the oligomeric/polymeric adducts formed by the reaction of LSL with HDI. In addition to this, low molecular weight fractions can be detected (a and b Peaks in Figure 9), which correspond to residual HDI and to mono- and diurethanes from quenching residual HDI with excessive MeOH. Reducing the index to 1.1 leads to a product mixture that is not completely soluble any more, indicating significant crosslinking as predicted by the Flory–Stockmayer (Equation (1)). SEC analysis of the soluble fraction proves crosslinking/branching to a substantial extent (Figure 9, dashed line). Inverting the NCO/OH ratio to an index of 0.5 leads to a product with incomplete crosslinking (Figure 9, solid line). This appears to be contradicting the prediction for the lower limit of 0.33 using the Flory–Stockmayer relation. Presumably, unreacted OH groups are sterically shielded by vicinal urethane groups formed first. This reduces the effective amount of OH groups for the reaction with HDI. Consequently, the effective lower limit of the Flory–Stockmayer gelation range is somewhat increased and seems to be larger than 0.5.

##### Kinetic Investigations

**Figure 10:**
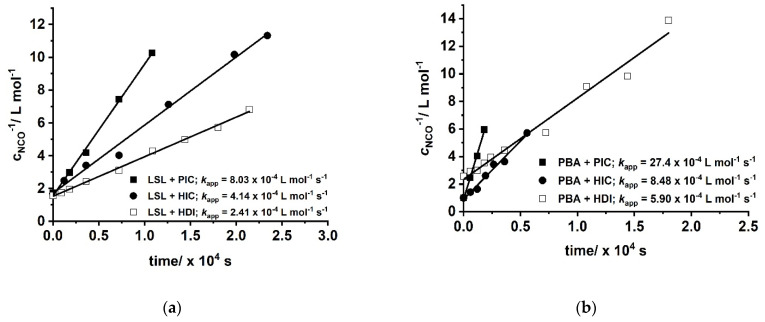
Second order kinetic plots of reactions of PIC, HIC, and HDI with (**a**) LSL; (**b**) commercially available polyester PBA. Reaction conditions: solvent = acetone; T = 50 °C; c_DBTDL_ = 500 ppm; index = 1.1.

**Figure 11:**
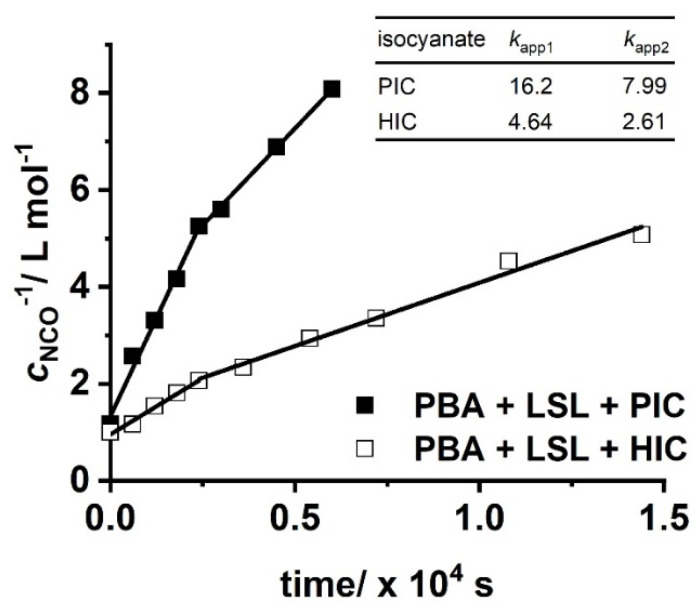
Second order kinetic plots of reactions of PIC and HIC with an equimolar mixture of LSL and PBA. Reaction conditions: solvent = acetone; T = 50 °C; c_DBTDL_ = 500 ppm; index = 1.1. Rate constants *k*_app_ shown in the inlet are in ×10^−4^ L mol^−1^ s^−1^.

The reaction between OH and NCO groups is generally accepted to follow second order kinetics [36,82] and can be studied by investigation of the corresponding second order plot and determination of *k*_app_ according to Equation (2) [38].

(2) $$c_{NCO}{}^{-1}=k_{app}\;t+{{c_{NCO}}_{0}}^{-1}$$

with $c_{NCO}=$ concentration of NCO groups at time *t* and $c_{NCO_{0}}=$ concentration of NCO groups at the beginning of the reaction. Figure 10 shows second order kinetic plots of reactions of PIC, HIC, and HDI with LSL and PBA, respectively. The relative reactivity of LSL and PBA towards the isocyanates is similar and follows the order PIC > HIC > HDI. All determined *k*_app_ are in the typical range for rate constants of conversions of isocyanates with primary or secondary alcohols [83]. In fact, PBA reacts with the corresponding isocyanates about 2.5 to 3.5 times faster than LSL (compare *k*_app_ in Figure 10a with the corresponding *k*_app_ in Figure 10b). This is because PBA is terminated by primary OH groups, whereas LSL contains secondary OH groups only. The difference in the reactivity is also observable in the second order plots of ternary reaction mixtures comprising isocyanate and an equimolar mixture of PBA and LSL (Figure 11). From this, two different *k*_app_ for each reaction can be calculated being *k*_app1_ = 16.2 × 10^−4^ L mol^−1^ s^−1^ and *k*_app2_ = 7.99 × 10^−4^ L mol^−1^ s^−1^ for the PBA/LSL/PIC mixture and *k*_app1_ = 4.64 × 10^−4^ L mol^−1^ s^−1^ and *k*_app2_ = 2.61 × 10^−4^ L mol^−1^ s^−1^ for the PBA/LSL/HIC reaction. The differences between *k*_app1_ and *k*_app2_ is about a factor of two in each ternary system, suggesting that LSL and PBA do not affect each other in terms of reactivity towards isocyanates.

##### Behavior of Ternary Systems

**Figure 12:**
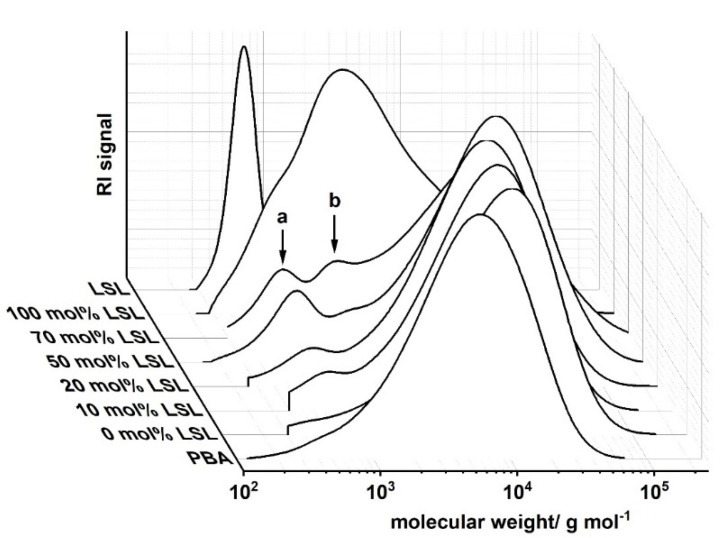
Size exclusion chromatograms of products from the reaction of HDI with LSL/PBA polyol mixtures comprising different molar LSL fractions. Reaction conditions: solvent = acetone; T = 50 °C; c_DBTDL_ = 500 ppm; index = 0.5. Peak **a** corresponds to residual LSL, peak **b** to LSL-HDI-LSL trimer.

**Figure 13:**
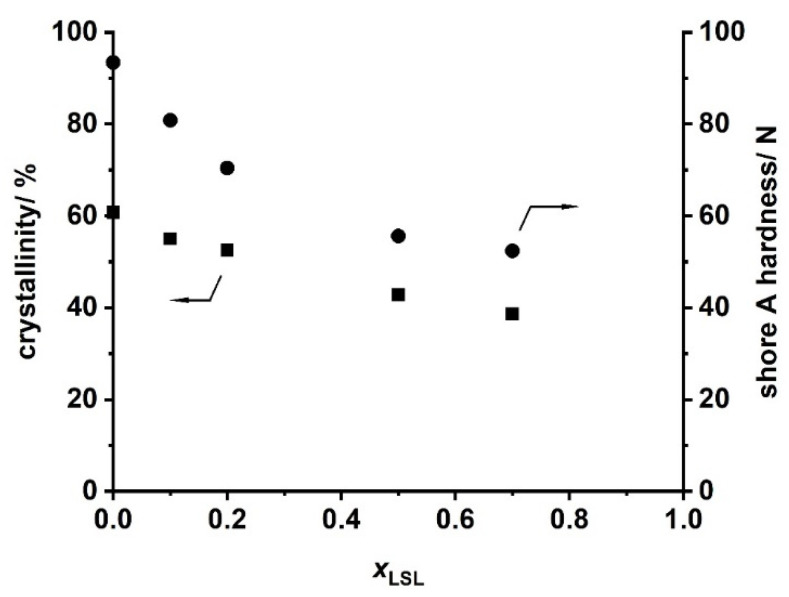
Crystallinity obtained by XRD and shore A hardness of products from the reaction of HDI with LSL/PBA polyol mixtures comprising different molar LSL fractions (x_LSL_). Reaction conditions: solvent = acetone; T = 50 °C; c_DBTDL_ = 500 ppm; index = 0.5.

Ternary PU systems containing LSL, PBA, and HDI are well-suited model formulations to study the influence of LSL on the product performance. For this, the content of LSL is systematically varied in the polyol mixture. To prevent significant crosslinking and enable reasonably high molecular weights, an index of 0.5 is applied. SEC analysis reveals a monomodal molecular weight distribution for the binary system PBA/HDI (0 mol.% LSL) with an average molecular weight of M_n_ = 4820 g mol^−1^, which agrees well to M_n,theo_ of 4619 g mol^−1^ according to Flory (Figure 12) [84]. In contrast to that, increasing the amount of LSL in the polyol mixture leads to a broadening of the molecular weight distribution and an increase of modality. The SECs of the samples containing 70 mol % and 50 mol % LSL in particular reveal signals at molecular weights that correspond to pure LSL. This strongly suggests incomplete incorporation of LSL in the PU system. This is most likely because PBA is consumed first due to the reduced reactivity of the secondary OH groups of LSL. Consequently, residual LSL remains in the reaction mixture. An increased amount of LSL in the product leads also to a reduction of properties such as shore A hardness and crystallinity, suggesting a plasticizing character of LSL (Figure 13).

### 3.2. (ω-1) HFA from LSL as Polyol Component for PU Systems

#### 3.2.1. (*ω*-1) HFA-Based Polyester Polyols and PU Systems Thereof

**Figure 14:**
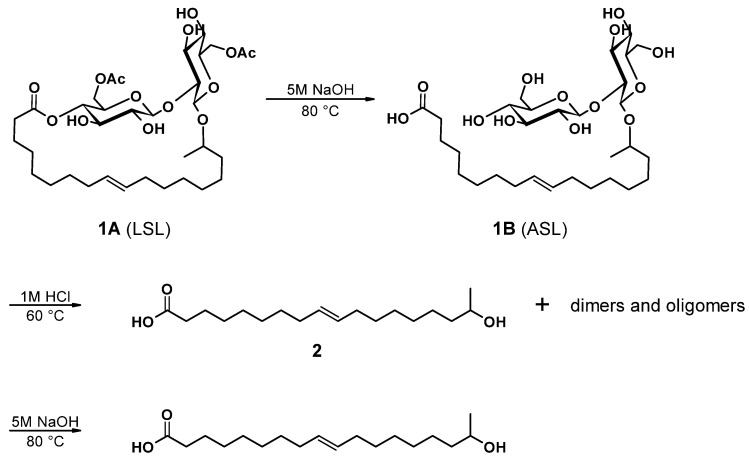
Synthesis route to (*ω*-1) hydroxyl fatty acids ((**2**), HFA) starting from the lactonic form of sophorolipid ((**1A**), LSL) via intermediate ((**1B**), ASL). (See text for yields).

The preparation of (*ω*-1) HFA (**2**) proceeds according to the sequence shown in Figure 14. Successive alkaline and acidic hydrolysis lead to a mixture of monomeric HFA with its dimers and some oligomers up to an overall yield of 74.5%. Further purification to produce pure monomeric HFA is possible by repeated saponification and solvent extraction, but reduces the overall yield to 48.4%. Fortunately, the subsequent polymerization towards A/B-type polyesters does not require highly pure monomeric HFA, since the dimers condense in the same way and yield the desired A/B-type polyesters. The further polymerization is thus investigated, applying the mixture of monomeric and dimeric/oligomeric (*ω*-1) HFA.

OH-terminated A/B-type polyesters can be produced by modifying a procedure published elsewhere [61]. In this synthesis, the mixture of monomeric and dimeric/oligomeric (*ω*-1) HFA is polymerized in presence of 1,6-hexanediol. The product is highly viscous, sticky, and not completely soluble in THF (M_n_ of soluble fraction ≈ 2200 g mol^−1^). For comparison, a polyester consisting of 12-hydroxystearic acid and 1,6-hexanediol is produced applying equal conditions. This product is oily and highly viscous as well; however, remains fully soluble in THF (M_n_ ≈ 2600 g mol^−1^). These outcomes support other investigations [81], which indicate that notable crosslinking has occurred during the synthesis of (*ω*-1) HFA-based polyester diols and is most likely the result of inseparable glucose impurities remaining in the (*ω*-1) HFA.

The reaction of HDI with the soluble fraction of (*ω*-1) HFA-based polyester diols yields a dark brownish oily product that is not completely soluble in THF. IR and SEC analysis reveals the product to be PU with M_n_ *≈* 3500 g mol^−1^ and a significant amount of species with M_n_ >10^6^ g mol^−1^ (results not shown). This is also attributed to residual glucose moieties leading to notable crosslinking and branching during PU synthesis.

#### 3.2.2. Direct Conversion of (*ω*-1) HFA to PU by Curtius Rearrangement

**Figure 15:**
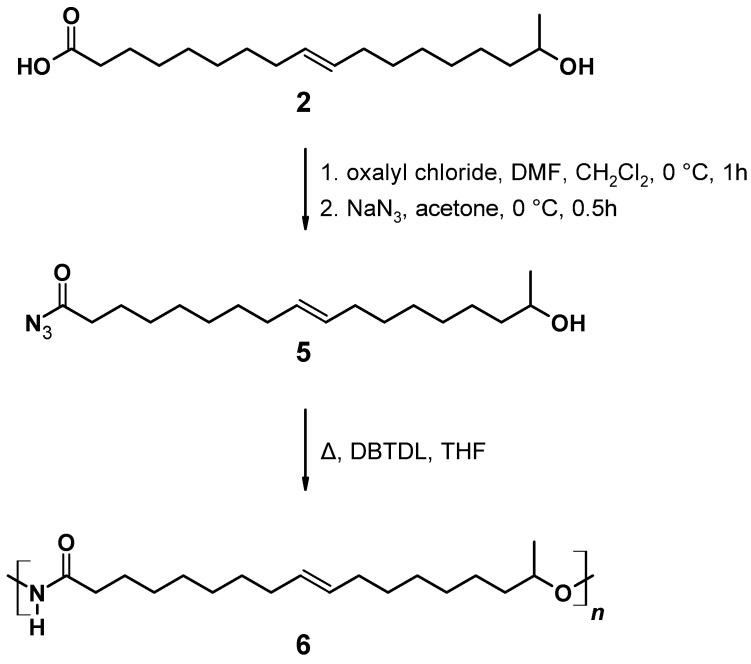
Reaction scheme for the reaction of (*ω*-1) HFA (**2**) producing (*ω*-1) HFA azide (**5**) and successive A/B type polymerization via Curtius rearrangement.

**Figure 16:**
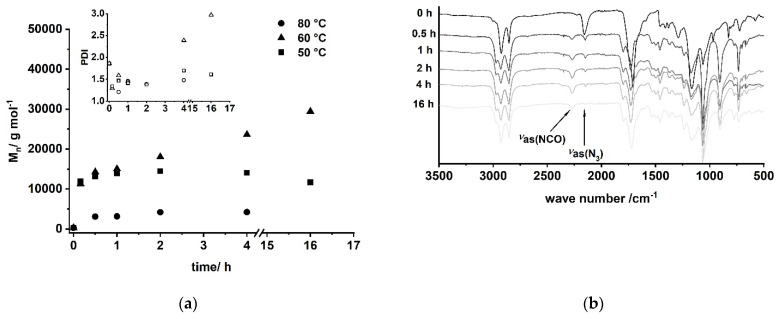
(**a**) Evolution of molecular weight and PDI of A/B type polymerization of (*ω*-1) HFA azide (**5**) via Curtius rearrangement determined by SEC; (**b**) reaction monitoring of the polymerization by FT-IR spectroscopy for T = 60 °C.

A/B type PU (**6**) can be obtained by Curtius rearrangement [51,62,63,64,65,66,67,68], thus, fatty acid derivatives such as (*ω*-1) HFA represent potential A/B type monomers for the synthesis of PU [69]. By modification of model reactions [72,73], a reaction procedure comprising (*ω*-1) HFA (**2**) → (*ω*-1) HFA azide (**5**) → (*ω*-1) HFA-based A/B PU (**6**) could be developed (Figure 15). This sequence leads to macromolecular species with M_n_ of max. 30,000 g mol^−1^ after extended reaction time (Figure 16a). With a reaction temperature of 60 °C, the product obtained after 16 h shows a notably high PDI that indicates significant side reactions such as crosslinking, branching, or the formation of macrocycles. Reduction of the reaction temperature to 50 °C leads to species with M_n_ of about 15,000 g mol^−1^ and PDIs in the range between 1.5 and 2.0. An increase of the temperature to 80 °C leads to a significant reduction of obtained molecular weight most likely because of favored formation of macrocycles [51,69].

FT-IR reaction monitoring of the polymerization step clearly shows the typical strong asymmetric stretching frequency of triatomic N_3_ groups at 2150 cm^−1^ for the starting material (Figure 16b) [85]. The reaction signals at 1795, 1720, and 1540 cm^−1^ become distinct after 16 h, indicating the formation of the urethane functionality [86,87,88,89]. In addition to these signals, the asymmetric stretch vibration of the NCO group [85,86,90,91] at 2264 cm^−1^ becomes evident after 30 min, suggesting effective decomposition of the azide. Note that this signal is still observable after 16 h reaction time, indicating incomplete conversion and most likely the formation of NCO-terminated species.

## 4. Conclusions

In contrast to acetic and deacetylated sophorolipid (ASL), the lactonic and acetylated form of sophorolipid (LSL) is soluble in solvents commonly used in PU synthesis. The conversion of LSL with mono- or diisocyanates is feasible and the products obtained show the expected behavior for PU systems based on polyols with functionalities higher than two, i.e., crosslinking in the reaction with diisocyanates applying indices below the point of gelation. The reactivity of LSL is in the typical range for conversions of isocyanates with secondary alcohols and about two to three times lower compared to polyols containing exclusively primary OH end groups such as commercially available PBA. This difference leads to incomplete incorporation of LSL in, e.g., ternary HDI/PBA/LSL systems applying an index of 0.5. Consequently, LSL acts here like a plasticizer reducing for instance crystallinity and shore A hardness. (*ω*-1) HFA-based polyester polyols are in general producible; however, low amounts of residual glucose impurities seem to lead to considerable crosslinking during the synthesis. Subsequent reaction of soluble fractions of this polyester with HDI leads to different PU products with M_n_ of about 3500 g mol^−1^ and >10^6^ g mol^−1^, respectively, indicating substantial crosslinking and branching. (*ω*-1) HFA-based A/B type PU systems are also feasible by applying a reaction procedure comprising (*ω*-1) HFA (**2**) → (*ω*-1) HFA azide (**5**) → (*ω*-1) HFA-based A/B PU (**6**). Such systems show molecular weights up to ca. 30,000 g mol^−1^. The products obtained show a significant amount of NCO groups remaining in the system, suggesting incomplete conversion and formation of NCO-terminated PU species.

## Figures and Tables

**Table 1:** Conditions for the reaction of LSL or PBA with 1-isocyanatohexane (HIC), phenylisocyanate (PIC) or 1,6-hexamethylenediisocyanate (HDI) in acetone.

Sample	Polyol	Isocyanate	n_NCO_/mol	n_NCO_/n_OH_	c_DBTDL_/ppm ^1^	T/°C
LSL−HIC−S1	LSL	HIC	0.0317	1.1	125	30
LSL−HIC−S2	LSL	HIC	0.0317	1.1	125	50
LSL−HIC−S3	LSL	HIC	0.0317	1.1	500	50
LSL−HIC−S4	LSL	HIC	0.0432	1.5	500	50
LSL−PIC−S1	LSL	PIC	0.0317	1.1	125	30
LSL−PIC−S2	LSL	PIC	0.0317	1.1	125	50
LSL−PIC−S3	LSL	PIC	0.0317	1.1	500	50
LSL−PIC-−S4	LSL	PIC	0.0432	1.5	500	50
LSL−PIC−S5	LSL	PIC	0.1440	5.0	500	50
LSL−HDI_0.5_	LSL	HDI	0.0072	0.5	500	50
LSL−HDI_1.1_	LSL	HDI	0.0158	1.1	500	50
LSL−HDI_10_	LSL	HDI	0.1440	10.0	500	50
PBA + PIC	PBA	PIC	0.0220	1.1	500	50
PBA + HIC	PBA	HIC	0.0220	1.1	500	50
PBA + HDI	PBA	HDI	0.0048	1.1	500	50

^1^ relative to n_OH_.

**Table 2:** Conditions to produce ternary PU systems based on LSL and PBA. (solvent = acetone, T = 50 °C, c_DBTDL_ = 500 ppm, x_LSL_ = molar fraction of LSL, T_M_ = melting point).

Sample	x_LSL_	n_LSL_/mol	n_PBA_/mol	n_NCO_/mol	T_M_/°C
PBA−HDI−0%LSL	0.0	-	0.0222	0.0110	47.51
PBA−HDI−10%LSL	0.1	0.0022	0.0200	0.0122	41.39
PBA−HDI−20%LSL	0.2	0.0050	0.0200	0.0150	40.65
PBA−HDI−50%LSL	0.5	0.0178	0.0178	0.0263	42.57
PBA−HDI−70%LSL	0.7	0.0100	0.0235	0.0285	-
PBA−HDI−100%LSL	1.0	0.0073	-	0.0073	46.22 ^1^

^1^ T_g_.

**Table 3:** Visually evaluated solubility of lactonic diacetylated sophorolipid (**1A**) (LSL) and acidic deacetylated sophorolipid (**1B**) (ASL) in selected solvents sorted by increasing solvent polarity (normalized molar transition energy ($E_{T}^{N}$) values [77,78]). (n_LSL_ = n_ASL_ = 0.145 mmol; solvent volume = 2 mL; + = soluble, − = not soluble).

Solvent	$E_{T}^{N}/\mathrm{kJ}\;{\mathrm{mol}}^{-1}$ [77,78]	Solubility LSL	Solubility ASL
Petroleum ether	− ^1^	−	−
Cyclohexane	0.025	−	−
*n*-Hexane	0.038	−	−
Toluene	0.414	+	−
Diethyl ether	0.490	−	−
*tert*-Butyl methyl ether (MTBE)	0.519	−	−
1,4-Dioxane	0.687	+	−
Tetrahydrofuran (THF)	0.867	+	−
Ethyl acetate	0.955	−	−
Chloroform	1.084	+	−
Dichloromethane	1.294	+	−
2-Butanone (MEK)	1.369	+	−
Acetone	1.486	+	−
N-Methylpyrrolidin-2-one (NMP)	1.486	+	−
N,N-Dimethylformamide (DMF)	1.616	+	+
Dimethyl sulfoxide (DMSO)	1.859	+	+
Acetonitrile	1.926	+	−
2-Propanol	2.286	−	−
Ethanol	2.738	−	−
Methanol	3.190	+	+
Water	4.187	+	+

^1^ not listed.
